# Recent US Case of Variant Creutzfeldt-Jakob Disease—Global Implications

**DOI:** 10.3201/eid2105.142017

**Published:** 2015-05

**Authors:** Atul Maheshwari, Michael Fischer, Pierluigi Gambetti, Alicia Parker, Aarthi Ram, Claudio Soto, Luis Concha-Marambio, Yvonne Cohen, Ermias D. Belay, Ryan A. Maddox, Simon Mead, Clay Goodman, Joseph S. Kass, Lawrence B. Schonberger, Haitham M. Hussein

**Affiliations:** Baylor College of Medicine, Houston, Texas, USA (A. Maheshwari, A. Parker, A. Ram, C. Goodman, J.S. Kass);; Harris Health System, Houston (A. Maheshwari, A. Parker, A. Ram, J.S. Kass);; Texas Department of State Health Services, Austin, Texas, USA (M. Fischer);; Case Western Reserve University School of Medicine, Cleveland, Ohio, USA (P. Gambetti, Y. Cohen);; University of Texas Medical School at Houston, Houston (C. Soto, L. Concha-Marambio);; Universidad de los Andes, Santiago, Chile (L. Concha-Marambio);; Centers for Disease Control and Prevention, Atlanta, Georgia, USA (E.D. Belay, R.A. Maddox, L.B. Schonberger);; University College London Institute of Neurology, London, UK (S. Mead);; HealthPartners Clinics & Services, St. Paul, Minnesota, USA (H.M. Hussein)

**Keywords:** Prions and related diseases, mad cow disease, bovine spongiform encephalopathy, pulvinar sign, PMCA, cortical ribbon sign, United States, United Kingdom, global health, Creutzfeldt-Jakob disease

## Abstract

Variant Creutzfeldt-Jakob disease (vCJD) is a rare, fatal prion disease resulting from transmission to humans of the infectious agent of bovine spongiform encephalopathy. We describe the clinical presentation of a recent case of vCJD in the United States and provide an update on diagnostic testing. The location of this patient’s exposure is less clear than those in the 3 previously reported US cases, but strong evidence indicates that exposure to contaminated beef occurred outside the United States more than a decade before illness onset. This case exemplifies the persistent risk for vCJD acquired in unsuspected geographic locations and highlights the need for continued global surveillance and awareness to prevent further dissemination of vCJD.

## Abstract 2

A recently diagnosed case highlights the need for continued global surveillance.

Prion disorders are a unique class of diseases caused by pathologically misfolding proteins leading to neurodegeneration (*1*). Creutzfeldt-Jakob disease (CJD), one such prion disorder, is divided into 4 etiologic categories: sporadic (sCJD), familial, iatrogenic, and variant (vCJD). CJD is incurable and inevitably fatal.

The emergence of vCJD was linked to an earlier epidemic of bovine spongiform encephalopathy (BSE) in the United Kingdom through the consumption of contaminated beef (*2*). Since the first report in 1996, a total of 229 vCJD cases have been reported worldwide: 177 in the United Kingdom; 27 in France; and 25 distributed in 10 other countries, including the United States (*3*). Incidence of vCJD peaked in the United Kingdom and France in 1999 and 2004, respectively. Countries outside of the United Kingdom were apparently affected through importation of beef and live cattle from the United Kingdom (*3*,*4*). Only 5 vCJD cases have previously been reported in North America: 3 in the United States and 2 in Canada. All 5 patients had lived in either the United Kingdom (3 patients) or the Kingdom of Saudi Arabia (2 patients), prompting the conclusion that vCJD was acquired overseas rather than in the United States or Canada (*5*).

We report the fourth confirmed case of vCJD in the United States and note that, unlike for the previous 3 cases, the specific country in which this BSE/vCJD infection was acquired is less clear. We found no definite epidemiologic link to a country where other known vCJD patients probably had been infected. We also review the clinical features and diagnostic challenge of vCJD, with special consideration of the possible public health concerns and global risks inherent to this disease.

## The Patient

The patient was a man in his forties who was born and raised in the Middle East. He lived in Russia for several years while completing a professional degree and briefly returned to the Middle East before taking up his final residence in the United States during the late 1990s.

Family members, friends, and co-workers were interviewed. The Texas Department of State Health Service’s investigation ascertained and verified that he had resided in Lebanon, Kuwait, Russia, and the United States. The patient’s family affirmed that, if the man had traveled to European countries, that travel was brief and infrequent. We found no evidence indicating that he had ever stayed in Great Britain, France, Ireland, or Saudi Arabia.

**Figure 1:**
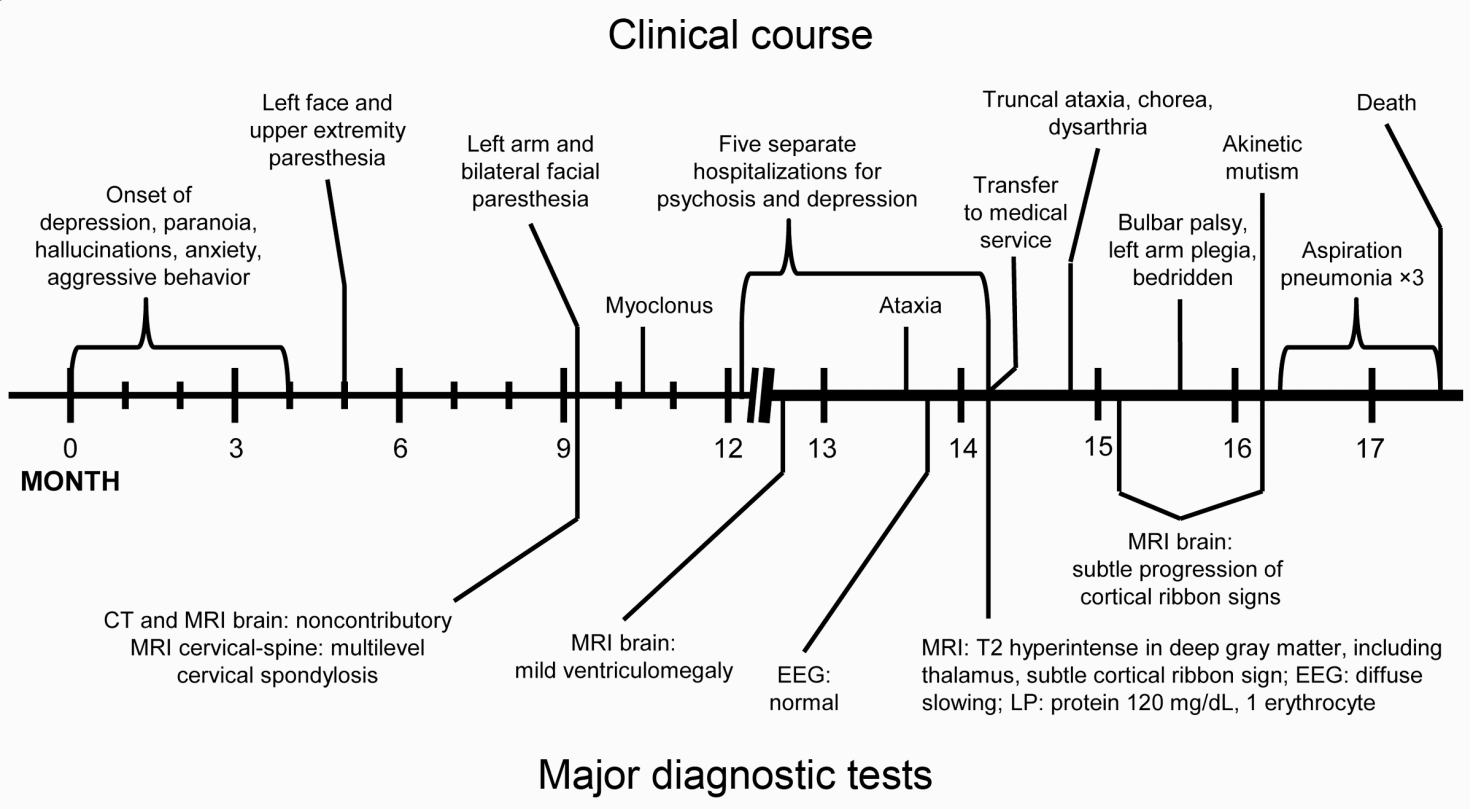
Timeline of course of illness and major diagnostic tests for US patient with variant Creutzfeldt-Jakob disease. CT, computed tomography; EEG, electroencephalography; MRI, magnetic resonance imaging; LP, lumbar puncture..

The patient’s family and co-workers described him as healthy and high-functioning before illness onset. The condition first manifested in late 2012 as depression and anxiety. These symptoms were initially subtle and did not interfere with his daily activities. Delusions and hallucinations were observed shortly after the onset of illness and in subsequent months were associated with changes in behavior in the form of withdrawal, isolation, secrecy, aggression, poor judgment, and lack of insight (Figure 1).

Intermittent numbness and paresthesias of the left face and upper extremity began in the fifth month of illness. These symptoms were attributed to a motor vehicle collision within the previous weeks. A few months later, when the symptoms started to affect the contralateral side, brain and cervical spine magnetic resonance imaging (MRI) was performed and showed multilevel degenerative changes and spinal foraminal stenosis, leading to the conclusion that most of his signs and symptoms were caused by cervical disc disease. The etiology for his facial paresthesias remained unclear.

The patient’s psychiatric condition gradually continued to worsen, and he was hospitalized multiple times in the 13th and 14th months after illness onset. Several diagnoses were entertained, including depression with psychotic features and bipolar disorder with psychosis. Despite treatment of symptoms, his condition continued to deteriorate.

**Figure 2:**
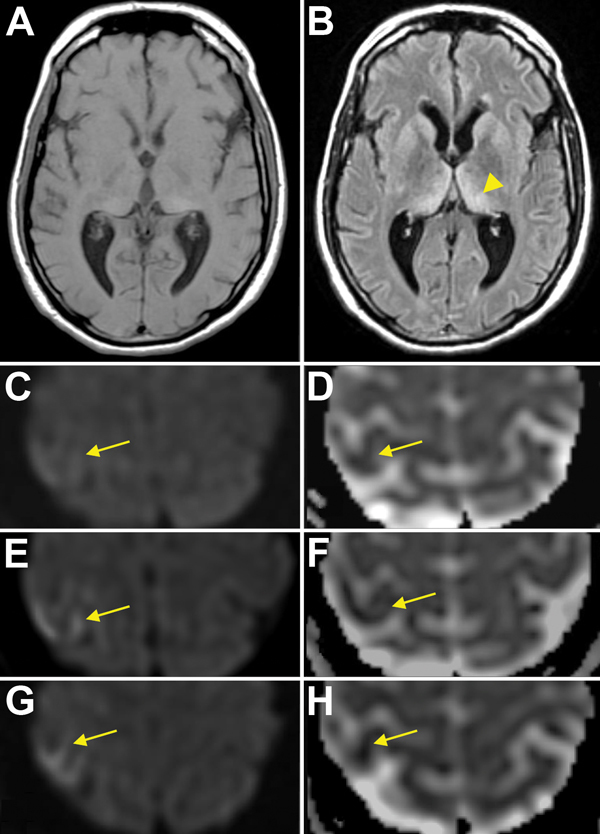
Magnetic resonance imaging (MRI) results for a US patient with variant Creutzfeldt-Jakob disease. T1 sequence (A) and T2 FLAIR sequence (B) show the “pulvinar” or “hockey stick” sign (arrowhead). Initial diffusion weighted imaging (DWI) (C) and apparent diffusion coefficient (ADC) (D) images show subtle restricted diffusion in the right primary motor cortex (arrows). Subsequent MRIs show increasing hyperintensity (arrows) on DWI (E, then G) and further attenuation (arrows) on ADC (F, then H), consistent with the “cortical ribbon” sign.

Because of increasing agitation, the patient was once again brought to the emergency department in the 14th month after symptom onset, where the neurology service was consulted. He was restless, irritable, disinhibited, and impulsive. He exhibited choreiform movements, most pronounced in the left upper extremity, in addition to myoclonus and ataxia. Reflexes were reduced throughout the upper and lower extremities. MRI of the brain showed mild diffuse volume loss, T2 hyperintensity with subtle restricted diffusion in the pulvinar nuclei of bilateral thalami (pulvinar sign), and subtle restricted diffusion in the right frontal cortex (cortical ribbon sign) (arrows) (Figure 2). Analysis of cerebrospinal fluid (CSF) showed elevated protein at 120 mg/dL (reference range 15–60 mg/dL) and a normal cell count, prompting a course of intravenous steroids and plasmapheresis for suspected autoimmune encephalitis.

Extensive investigations for infectious and autoimmune encephalitides, malignancy, heavy metal intoxication, vitamin deficiency, and rheumatologic and endocrine disorders were concomitantly pursued. CSF was sent to the National Prion Disease Pathology Surveillance Center (NPDPSC; Cleveland, OH, USA) for evaluation of 14-3-3 and Tau protein levels, as well as real-time quaking-induced conversion (RT-QuIC) testing for prion detection (Table 1).

**Table 1:** Relevant studies for the diagnosis of vCJD, United States*

Test†	Result (reference range)
Blood‡	
Albumin, g/dL	2.3–4.1 (3.4–5.0)
Ammonia, μmol/L	31 (11–32)
Anti-thyroglobulin antibody, IU/mL	<1.0 (0.0–0.9)
Anti-thyroid peroxidase antibodies, IU/mL	5 (0–34)
Ceruloplasmin, mg/dL	32.5 (20.0–60.0)
Creatine kinase, U/L	39–257 (0.6–1.3)
C-reactive protein, mg/dL	0.098 (0.0–0.3)
Erythrocyte sedimentation rate, mm/h	7 (0–15)
Ethanol level, g/dL	Undetectable (0.0–0.08)
Hemoglobin A1c, %	5.0 (4.3–6.1)
Protein, g/dL	5.1–7.8 (6.4–8.2)
Rapid plasma reagin	Nonreactive
Toxoplasma IgG, IU/mL; IgM, AU/mL	<3.0, <3.0 (<5.9, <7.9)
Thyroid stimulating hormone, U/mL	1.78 (0.36–3.74)
Vitamin B1, nmol/L	254.5 (66.5–200.0)
Vitamin B12, pg/mL	597–926 (211–911)
Cerebrospinal fluid§	
Electrophoresis	No oligoclonal bands
Glucose, mg/dL	46–53 (50–80)
Protein, mg/dL	90.0–204.2 (15–45)
Erythrocytes, cells/μL	1–2 (0)
Leukocytes, cells/μL	1–3 (0–5)
Angiotensin converting enzyme, U/L	1.5 (0.0–2.5)
Epstein-Barr virus PCR	Not detected
Herpes simplex viruses 1 and 2	Not detected
VDRL	Nonreactive
Stain	No organisms found
Culture	No growth
Other diagnostic tests	
Electroencephalography x3	Mild to moderate diffuse slowing; no epileptiform activity
MRI brain	14 mo after initial symptoms: bilateral T2 hyperintensities in the thalamic pulvinar nuclei and, to a lesser extent, in the caudate and lentiform nuclei. Subtle cortical ribbon sign over the right motor cortex
	At 15 mo: persistent pulvinar and cortical ribbon sign; resolution of caudate and lentiform nuclei T2 hyperintensities
	At 16 mo: persistent pulvinar and cortical ribbon sign, with interval development of subtle T1 hyperintensities
MRI, C/T/L-spine	Cervical and lumbar spondylosis; no cord compression
CT angiogram head and neck	No intracranial vascular abnormalities; mild large vessel atherosclerotic disease without significant stenosis
CT chest/abdomen/ pelvis; scrotal ultrasound:	No malignancy detected
CJD-specific laboratory tests	
Blood	
*PRNP* genotype	Codon 129 methionine homozygous; otherwise no mutations
Direct detection assay	Negative
Urine PMCA	Positive for scrapie prion protein
Cerebrospinal fluid	
14-3-3 protein	Negative
Tau protein, pg/mL	358, negative
RT-QuIC	Negative

*Includes tests that rule out vCJD mimics. Routine serum electrolytes and cell counts were otherwise normal. CJD, Creutzfeldt-Jakob disease; CT, computed tomography; MRI, magnetic resonance imaging; PMCA, protein misfolding cyclic amplification; RT-QuIC, real-time quaking-induced conversion; vCJD, variant CJD; VDRL, Venereal Disease Research Laboratory. †Urinalyses were negative for heavy metals, drug toxicity, and copper. ‡Blood tests for antinuclear antibody, antineuronal nuclear antibody (ANNA1, Anti-Hu antibody), anti-Purkinje cell antibody (anti-Yo antibody), anti-smooth muscle/ribonucleoproteins; anti-Sjögren’s-syndrome-related antigen A, and anti-Sjögren’s-syndrome-related antigen B, *Aspergillus* antibody, *Blastomyces* antibody, *Coccidiodes* antibody, hepatitis panel, HIV-1/HIV-2, and Borrelia burgdorferi PCR were all negative. §Negative for cryptococcal antigen; IgM against West Nile virus, St. Louis encephalitis virus, California encephalitis virus, eastern equine encephalitis, western equine encephalitis virus, and West Nile virus; fluorescent treponemal antibody; and autoimmune/paraneoplastic panel (Dalmau).

During the first several weeks after admission, the patient’s behavior and chorea improved, which was interpreted as a possible response to immunomodulatory therapy. He therefore underwent further intravenous immunoglobulin treatment and received 2 doses of rituximab. After a few weeks of a relatively stationary course, his condition deteriorated. Dysarthria, truncal ataxia, and lower extremity weakness developed, with subsequent loss of his ability to ambulate.

After the laboratory tests for antibody-mediated autoimmune and paraneoplastic encephalidites yielded negative results, “probable vCJD” was diagnosed. The Texas Department of State Health Service and the Centers for Disease Control and Prevention (Atlanta, GA, USA) were notified of the suspected case. Samples of blood, urine, and CSF were sent to the NPDPSC and the Medical Research Council Prion Unit (London, UK). Brain and tonsil biopsies were deferred because of concern that the patient would not tolerate either biopsy procedure.

By the 16th month of illness, left arm plegia and a severe bulbar palsy developed. Within 1 month, the patient became akinetic-mute and entirely bedridden. After the third episode of aspiration pneumonia, sepsis developed. After discussion with the family, goals of care were restricted to comfort measures only. The patient died shortly thereafter, almost 18 months after initial onset of symptoms.

## Results of Histopathologic Examination and Biochemical Tests

**Figure 3:**
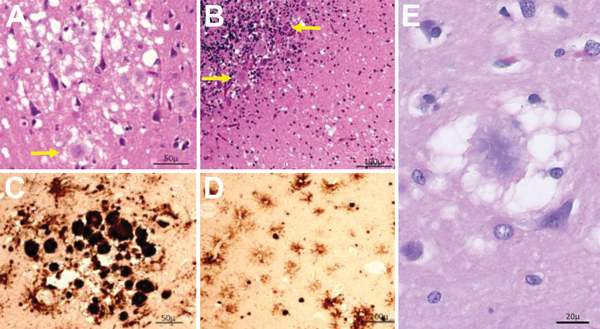
Results of histopathologic and immunohistochemical analyses for a US patient with variant Creutzfeldt-Jakob disease. A) Hematoxylin and eosin staining shows many typical florid plaques (A, arrow) occasionally forming clusters; large vacuole spongiform change is also present (original magnification ×10). B) Plaques often not of the florid type along with spongiform change are present in cerebellum (arrows; original magnification ×20). C, D) Prion protein immunostaining confirms the presence of PrP in the plaques, which intensely immunostained (C; original magnification ×10), and highlights small round cells surrounded by short processes (D, antibody 3F4; original magnification ×40). E) Under high magnification, a prion plaque with spongiform change is seen in the left frontal cortex (original magnification ×400).

A brain-only autopsy was performed. Conventional histologic examination demonstrated numerous typical florid plaques that occasionally formed clusters and were often mixed with patches of spongiform change made of large vacuoles (Figure 3, panel A). This lesion pattern was present throughout the cerebral cortex, except for the hippocampal formation and lower temporal gyri. Basal ganglia and thalamus showed severe spongiform change with only scattered plaques. Prion plaques, but not well-formed florid plaques, were present in the granule cell layer of the cerebellum (Figure 3, panel B).

Prion protein immunostaining showed intense immunoreaction of the core of the florid plaques and patchy and granular deposits that were arranged in rounded clusters (Figure 3, panel C). In addition, the immunostaining highlighted cells with short processes stemming from the round perikaryon in a spoke wheel–like fashion (Figure 3, panel D). These cells were observed especially in the cerebral cortex and molecular layer of the cerebellum, where plaques and plaque-like deposits were also present. Clusters of intensely staining kuru plaques were seen in the granule cell layer of the cerebellum.

**Figure 4:**
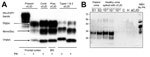
Results of biochemical testing of a US patient with variant Creutzfeldt-Jakob disease (vCJD). A) Immunoblot of vCJD patient and controls. All the PK-treated preparations show similar electrophoretic profiles characterized by 3 bands displaying different mobilities according to number of the linked sugar moieties. The samples from this case and another vCJD case used as positive control (third lane) show the overrepresentation of the diglycosylated band, whereas in sCJD cases, the monoglycosylated band is the most prominent. In both vCJD cases, the unglycosylated band co-migrates with type 2 (sixth lane) as indicated by the type 1 and 2 controls. The PK-untreated preparation (first lane) is used as control of PK digestion. Total brain homogenate, antibody 3F4. B) PrP^Sc^ detection in urine by protein misfolding cyclic amplification (PMCA). A urine sample from the patient was processed as previously described (*6*), and the supernatant fraction was run in duplicate (S1, S2). Samples were subjected to 96 PMCA cycles in the presence of 10% TgHuM brain homogenate, used as the substrate for PMCA. PrP^Sc^ signal was assessed by Western blot after PK digestion. As a positive control, vCJD brain homogenate was spiked at 10^−5^, 10^−7^, and 10^−9^ dilutions into urine from a healthy person and processed at the same time and in the same manner for PMCA. Lane C, PMCA-negative control (no sample); lane H, urine from a healthy person; sCJD, urine from an sCJD patient. CJD, Creutzfeldt-Jakob disease; Contr, control; MonoGly, monoglycosylated; NBH, normal brain homogenate without PK treatment used as an electrophoretic migration marker; Pres, present; PK, proteinase K; PrP^Sc^, scrapie prion protein; sCJD, sporadic CJD; Unglyc, unglycosylated.

Western blot showed the typical electrophoretic pattern of the protease-treated scrapie prion protein (PrP^Sc^) associated with vCJD. This pattern, commonly identified as 2B, is characterized by the 19 kDa molecular weight of the unglycosylated band of PrP^Sc^ after proteinase K digestion, unlike type 1, in which the size of the unglycosylated band is 21 kDa (Figure 4, panel A). In addition, the distribution of the PrP^Sc^ glycoforms differs; the diglycosylated form is the predominant form in vCJD, whereas the most represented band in sCJD is the monoglycosylated form. 

Urine samples from this patient were collected before death and subjected to protein misfolding cyclic amplification (PMCA) analysis. Samples were tested twice, and both times the assay results were positive (Figure 4, panel B). Therefore, on the basis of histopathologic and biochemical analysis, the diagnosis of vCJD in the present case is definitive.

## Discussion

When a rare disease manifests with a common symptom, that makes the diagnosis even more difficult. Unlike sCJD, which often presents with the relatively rare symptom of rapidly progressing dementia, vCJD usually presents with psychiatric symptoms, which are relatively common. Because of these difficulties, diagnosis is usually delayed. Development of other neurologic manifestations (e.g., chorea, ataxia) supports a diagnosis of sCJD or vCJD. Painful sensory complaints can be a particularly helpful clue pointing to vCJD because they are generally not reported with sCJD. In addition, vCJD onset occurs at a younger age than does sCJD (mean 26 vs. 65 years), and disease course of vCJD is longer than that of sCJD (mean 14 months vs. 4 months) (*7*,*8*). Despite the statistical differences in onset and duration, the distinction between sCJD and vCJD is not always clear (*9*). In 1 study, 13 sCJD patients with psychiatric presentation had clinical characteristics more similar to vCJD than sCJD (younger age at onset and longer survival), and depression was the most common presenting symptom (*10*). Regardless, in the patient reported here, clinical onset and duration of illness were typical of vCJD. The clinical differentiation between sCJD and vCJD is reflected by the World Health Organization diagnostic criteria (*7*,*8*,*11*,*12*) (Table 2).

**Table 2:** World Health Organization diagnostic criteria for vCJD and sCJD*

Diagnosis	vCJD	sCJD
Possible	Progressive psychiatric disorder lasting >6 mo with no alternate explanation; at least 4 of the following: early psychiatric symptoms, persistent pain and/or dysesthesia, ataxia, chorea/ dystonia/myoclonus, and dementia; EEG without periodic sharp wave complexes typical for sCJD	Progressive dementia <2 y duration (typically <6 mo); at least 2 of the following: myoclonus, visual or cerebellar disturbance, pyramidal or extrapyramidal dysfunction, akinetic mutism; EEG atypical (not showing periodic sharp wave complexes) or not done
Probable	Meets criteria for possible vCJD plus:	Meets criteria for possible sCJD plus:
	EEG not consistent with sCJD; and bilateral pulvinar high signal on MRI of brain OR progressive psychiatric disorder lasting >6 mo with no alternate explanation; and positive tonsil biopsy	Typical EEG findings (generalized periodic sharp wave complexes at ≈1 Hz); and/or positive 14-3-3 assay in CSF and clinical duration leading to death in <2 y
Definite	Neuropathologic confirmation of vCJD	Neuropathologic confirmation of sCJD

*See (*11,13*). CJD, Creutzfeldt-Jakob disease; CSF, cerebrospinal fluid; EEG, electroencephalography; MRI, magnetic resonance imaging; sCJD, sporadic CJD; vCJD, variant CJD.

Three well-known diagnostic tests can point the clinician to sCJD: MRI (diffusion restriction and T2 hyperintensity of the cortex and basal ganglia), electroencephalography (periodic sharp wave complexes), and CSF analysis (elevated 14-3-3/Tau levels) (*13*). In contrast, for vCJD, electroencephalography and CSF analysis do not show any specific changes. The only positive finding of diagnostic value in vCJD from these tests is the pulvinar sign on MRI, which is not entirely specific for vCJD and can be absent in up to 9% of cases, even after multiple MRIs (*14*). According to World Health Organization criteria, the only way to diagnose “definite vCJD” while the patient is living is a brain biopsy (*15*). In contrast to sCJD, lymphoid tissue in vCJD has a high detection rate of PrP^Sc^ (*16*), so tonsil biopsy has become an alternative to brain biopsy, with recent investigations showing high sensitivity and specificity (*17*).

The panel of diagnostic tests for CJD used for many years at the NPDPSC includes level determinations of 14-3-3 and Tau proteins in the CSF. These tests are highly sensitive for sCJD, the most common form of human prion disease, but not for vCJD. A negative 14-3-3 has a negative predictive value of 63%, and a negative Tau has a negative predictive value of 81% (*18*). If both tests are negative, the negative predictive value for vCJD rises to 84% (*18*), which leaves ≈3 of 20 patients with a false-negative result, as in the patient reported here.

RT-QuIC, a relatively new test available at the NPDPSC, can detect minute amounts of prions in CSF by amplifying PrP^Sc^ using recombinant PrP as substrate. If positive, RT-QuIC is very specific for diagnosing sCJD, but it has been reported to be negative in vCJD (*3*), as in the CSF sample of the patient reported here.

Analysis of the PrP gene coding regions demonstrating the presence of methionine homozygosity at codon 129 has been found in all cases of proven primary vCJD examined (*3*,*19*), including in the case reported here. However, because >40% of the population can be methionine homozygous, sequencing of PrP gene coding regions is helpful only in providing supportive evidence for increased susceptibility to prion disease (*20*).

One experimental test for vCJD is a blood-based direct detection assay that takes advantage of the high affinity of PrP^Sc^ for metal surfaces to capture and immunodetect the minute amounts of PrP^Sc^ expected to be present in the blood of vCJD patients (*21*). Application of this test on specimens collected from >5,000 persons did not indicate false-positive findings; however, the sensitivity reached only 71% (*22*) and was negative in the patient in our report.

Another new experimental test is based on PrP^Sc^ detection in urine. This test uses PMCA technology to amplify and then visualize traces of PrP^Sc^ that might be present in the patient’s urine and has nearly 93% sensitivity and 100% specificity (*6*). Although the high accuracy of the assay might decrease when larger cohorts are examined, this entirely noninvasive test is likely to be useful in screening for vCJD. This test was the only one conducted on a sample obtained before the death of the patient reported here that yielded a positive result. PrP^Sc^ was detected in this patient only after 1 round of 96 PMCA cycles, indicating that the amount of PrP^Sc^ in urine was higher than that in most of the other vCJD patients analyzed (*6*).

The neuropathology of vCJD is distinct from that of sCJD, and the classification system for neuropathologic subtypes of sCJD has been described in detail elsewhere (*23*–*25*). The diagnosis of vCJD was definitively confirmed in this case by the postmortem histologic and PrP^Sc^ examinations, which demonstrated the widespread presence of florid plaques, the typical electrophoretic profile of the PrP^Sc^ (*3*), and the PrP immunostaining demonstrating rounded cells surrounded by short delicate processes resulting in a feathery appearance (*26*). The identity of these cells remains to be determined.

This case highlights several diagnostic challenges presented by vCJD. Studies on a vCJD cohort from the United Kingdom have shown a mean interval of 2.5 months between clinical onset and the first medical examination. The overall average delay between onset and suspicion of vCJD is 8.9 months (*7*). This case met similar delays, despite the typical clinical presentation (*3*).

The significantly elevated protein concentration in the CSF in this patient, up to 204 mg/dL, was a confounder. CSF protein in vCJD is usually normal; the highest concentration previously reported was 90 mg/dL (*19*). We remain unsure about the source of this finding. The cortical ribbon sign on MRI, although a common finding in sCJD, was also not previously reported in vCJD (*14*).

Another major difficulty in this case was proving the diagnosis with a high level of certainty before death so the goals of care could be adjusted accordingly. Despite our clinical suspicion, results of all the initial laboratory tests were negative. The pulvinar sign, which is relatively sensitive and specific for vCJD (*14*), did not exclude other disorders that rarely have similar features, including Wernicke’s encephalopathy and inflammatory limbic encephalitis (*27*–*29*). By the time brain or tonsil biopsy was considered, the patient was considered unable to tolerate the procedure. The only positive test specific for vCJD in this patient was PrP^Sc^ detection in urine using the PMCA test. Unfortunately, the result became available only after his death.

Perhaps the most challenging aspect of this case is identifying the geographic location of the patient’s exposure to prions. The patient, despite being a US citizen, was born and raised outside of the Americas in 3 countries that were importing UK beef at a time (during 1980–1996) when it was at increased risk for BSE contamination. According to the 2010 US Census, US citizens born outside the Americas constituted <5% of the US population, and not all of this small subgroup would have resided in the United Kingdom or in countries that had imported UK beef during the increased risk period, a residency history common to the 5 previously identified North American vCJD patients (*30*).

Exportation of BSE-contaminating beef from the United Kingdom during 1980–1996 and/or BSE-infected live cattle during 1980–1990 most likely is the main cause of BSE exposure outside the United Kingdom (*4*). During 1980–1996, the patient in our report spent >6 years in 2 countries—Kuwait and Russia—to which UK beef was exported. During that time, he also lived <1 year in Lebanon. Although the size of the Kuwaiti population was a few magnitudes smaller, based on UK export data for 1980–1996, the same order of magnitude (≈2.5 × 10^3^ metric tons) of beef was exported to Kuwait and to the Soviet Union/Russia when this patient resided in those 2 countries (*4*; Her Majesty’s Revenue and Customs, Overseas Trade Statistics, Crops & Trade Branch, Analysis and Evidence Team, unpub. data). These data suggest that the patient’s risk of eating possibly BSE-contaminated UK beef would have been substantially greater during his stay in Kuwait than during his stay in Russia. The risk that he ate such meat was lower in Lebanon than in Russia, primarily because of his relatively short stay in Lebanon during 1980–1996 and the fact that >85% of the 1980–1996 UK beef was imported to Lebanon after he moved to Russia.

This patient lived in the United States for 14 years before vCJD developed. A >14-year incubation period can be consistent with the mean incubation period for vCJD: 11.6–16.7 years, estimated by published models of the vCJD epidemic (*31*–*33*). Each of these models predicts a skewed curve toward much longer incubation periods than the mean as the vCJD epidemic wanes over time, similar to other human prion disease outbreaks (*31*,*34*,*35*). In addition, each of these models reflects a strikingly younger distribution of vCJD patients in the United Kingdom and supports the published concept that susceptibility to the prion infection that will cause vCJD peaks among adolescents and declines rapidly with age thereafter (*36*). Given that this patient did not come to the United States until his late 20s, this age-dependent susceptibility factor favors a conclusion that he was infected before he moved to the United States.

We deemed surgical or transfusion routes of vCJD transmission to or from the patient to be unlikely because the only known surgical procedure he had undergone was a circumcision performed in Kuwait when he was ≈8–10 years of age, and he had no history of receiving or donating blood. A review of records at the Gulf Coast Regional Blood Center and the American Red Cross provided further evidence that he had not been a US blood donor.

Given the markedly declining incidence of vCJD globally, this patient is only the fourth patient worldwide confirmed to have this disease since the beginning of 2012; the other 3 were from the United Kingdom and France (*3*). This case underscores that the diagnosis of vCJD should not be dismissed if the patient has not resided in a country with a known endemic case of vCJD. Given the several decades’ long potential incubation periods estimated from epidemiologic modeling, the international occurrence of additional vCJD cases can be reasonably anticipated (*34*,*35*).

Furthermore, UK surveys of archived appendix tissues indicate an approximate prevalence of asymptomatic vCJD infection of 1 in 2,000 persons born during 1941–1985 (*37*). Depending on genetic subtype, one may harbor the pathogenic prion and never develop symptoms (*3*). However, because the agent is transmissible through blood transfusions, organ transplants, and surgical instrumentation, iatrogenic propagation of the disease remains a real possibility (*3*).

The detection of a definitive case of vCJD in the United States highlights the importance of continuing enhanced national human prion disease surveillance. According to the World Organisation for Animal Health, the BSE status of the 3 countries where the patient reported here resided during the critical exposure period was “undetermined,” suggesting the lack of a systematic BSE surveillance system.

The potential difficulty in making the clinical diagnosis in many patients with vCJD and the delay with which the disease is first suspected raises the concern that vCJD can be missed. The need for neuropathology expertise and advanced neuropathologic techniques is probably an important limiting factor in some parts of the world. The MRI examination, along with the newly developed blood and urine tests, are among the most helpful premortem tests to diagnose vCJD. A postmortem brain and lymphoreticular tissue autopsy examination remains critical to confirm the diagnosis.
